# Supplementary material for: Content-rich biological network constructed by mining PubMed abstracts
Source: BMC Bioinformatics. 2004 Oct 8;5:147. doi: 10.1186/1471-2105-5-147 (PMC528731; doi:10.1186/1471-2105-5-147)
Supplement: Additional File 2 — The original results of the above study (non-essential files are deleted to keep the file size under the limit set by BMC bioinformatics). [file 1471-2105-5-147-S2.bz2 › chilibotAdditionalFile2/dip05/231ID7931156E750/html/UL52_UL8.html]

 


 **UL52** and **UL8** 
  
Found 43 abstracts in PubMed, retrieved 05.  
 

 What does Google say? 
 PDF only 
| .edu only 

---

**Interactive relationship** (e.g. stimulation, inhibition, etc)

**Neutral relationship**- Two hybrid analysis of the interaction between the  **UL52**  and  **UL8**  subunits of the herpes simplex virus type 1 helicase primase.  Ref: 10501495 J Gen Virol, 1999
- In contrast, the  **UL52**   **UL8**  interaction gave a strong signal in the two hybrid system.  Ref: 10501495 J Gen Virol, 1999
- The interactions of the  **UL52**  protein with the  **UL8**  and UL5 proteins were analysed by using the yeast two hybrid system.  Ref: 10501495 J Gen Virol, 1999
- Deletion analysis showed that a 548 amino acid fragment of  **UL52**  amino acids 366 914 retains the ability to interact with  **UL8** .  Ref: 10501495 J Gen Virol, 1999

**Non-interactive relationship** (e.g. studied together, co-existance, homology, etc.)

- Herpes simplex virus type 1 expresses a heterotrimeric helicase primase, the subunits of which are encoded by the viral UL5,  **UL8**  and  **UL52**  genes.  Ref: 10501495 J Gen Virol, 1999
- We show that the HSV 1 UL5,  **UL8** , UL29, UL30, UL42, and  **UL52**  gene products along with the AAV Rep68 protein are sufficient to initiate replication on duplex DNA containing the AAV origins of replication, resulting in products several hundred nucleotides in length.  Ref: 11581393 J Virol, 2001
- Herpes simplex virus type 1 encodes a heterotrimeric helicase primase complex composed of the products of the UL5,  **UL52** , and  **UL8**  genes.  Ref: 11278618 J Biol Chem, 2001
- The results shown in this paper are consistent with the model that the five proteins, ICP8, UL5,  **UL8** , UL9, and  **UL52** , form a scaffold.  Ref: 12634381 J Virol, 2003
- The ordered assembly of the herpes simplex virus HSV type 1 replication apparatus leading to replication compartments likely involves the initial assembly of five viral replication proteins, ICP8, UL9, and the heterotrimeric helicase primase complex UL5  **UL8**   **UL52** , into replication foci.  Ref: 12634381 J Virol, 2003
- the heterotrimeric helicase primase complex, encoded by UL5,  **UL8** , and  **UL52**  genes, is believed to unwind duplex viral DNA at replication forks.  Ref: 11642367 Prog Nucleic Acid Res Mol Biol, 2001
